# Natural disease history of the D2*-mdx* mouse model for Duchenne muscular dystrophy

**DOI:** 10.1096/fj.201802488R

**Published:** 2019-04-01

**Authors:** Maaike van Putten, Kayleigh Putker, Maurice Overzier, W. A. Adamzek, Svetlana Pasteuning-Vuhman, Jaap J. Plomp, Annemieke Aartsma-Rus

**Affiliations:** * Department of Human Genetics, Leiden University Medical Centre, Leiden, The Netherlands;; † Department of Neurology, Leiden University Medical Centre, Leiden, The Netherlands

**Keywords:** pathology, fibrosis, calcification, muscle function, regeneration

## Abstract

The C57BL/10ScSn-*Dmd^mdx^*/J (BL10-*mdx*) mouse has been the most commonly used model for Duchenne muscular dystrophy (DMD) for decades. Their muscle dysfunction and pathology is, however, less severe than in patients with DMD, which complicates preclinical studies. Recent discoveries indicate that disease severity is exacerbated when muscular dystrophy mouse models are generated on a DBA2/J genetic background. Knowledge on the natural history of animal models is pivotal for high-quality preclinical testing. However, for BL10-*mdx* mice on a DBA2/J background (D2-*mdx*), limited data are available. We addressed this gap in the natural history knowledge. First, we compared histopathological aspects in skeletal muscles of young D2-*mdx*, BL10-*mdx*, and wild-type mice. Pathology was more pronounced in D2-*mdx* mice and differed in severity between muscles within individuals. Secondly, we subjected D2-*mdx* mice to a functional test regime for 34 weeks and identified that female D2-*mdx* mice outperform severely impaired males, making females less useful for functional preclinical studies. Direct comparisons between 10- and 34-wk-old D2-*mdx* mice revealed that disease pathology ameliorates with age. Heart pathology was progressive, with some features already evident at a young age. This natural history study of the D2-*mdx* mouse will be instrumental for experimental design of future preclinical studies.—Van Putten, M., Putker, K., Overzier, M., Adamzek, W. A., Pasteuning-Vuhman, S., Plomp, J. J., Aartsma-Rus, A. Natural disease history of the D2-*mdx* mouse model for Duchenne muscular dystrophy.

Duchenne muscular dystrophy (DMD) is an X-linked recessive disorder affecting around 1 in 5000 newborn males worldwide (1, 2). Patients with DMD manifest their first clinical symptoms at the age of 3–4 yr and generally become wheelchair dependent between the ages of 7 and 13 yr (3, 4). The ambulation period can be prolonged in many boys with DMD with early initiation of steroid treatment (5, 6). The terminal stage of the disease starts when patients require assisted ventilation by the age of around 20 yr, and patients usually die in the third or fourth decade due to respiration or cardiac failure (7).

DMD is caused by out-of-frame mutations in the *DMD* gene that disrupt the open reading frame, resulting in the synthesis of nonfunctional dystrophin protein (8, 9). This leads to a loss of the connection between the cytoskeleton and the extracellular matrix making muscle fibers more susceptible to contraction-induced membrane damage. As a result, the uncontrolled influx of calcium occurs, causing progressive degeneration of myofibers (10, 11). This pathologic process is accompanied by inflammation and fibrosis (12), leading to muscle wasting and loss of function.

The C57BL/10ScSn-*Dmd^mdx^*/J (BL10-*mdx*) mouse is the most frequently used mouse model for DMD. These mice harbor a nonsense mutation in exon 23 of the *Dmd* gene, which leads to a premature stop of protein translation and nonfunctional dystrophin, leading to the typical hallmarks of muscular dystrophy (13). However, even though BL10-*mdx* mice are deficient for dystrophin, the disease phenotype is much milder than that of patients with DMD. This can be explained by, among other things, vast differences in size and muscle loading plus length of growth phase between species and possible differences in capacity for muscle regeneration across species (14–16). Given the difference in disease pathology, translating therapeutic benefits obtained in BL10-*mdx* mice to the human situation should be done with caution (17). As such, the availability of a more severely affected mouse model that more closely recapitulates the disease pathology observed in patients with DMD would be instrumental and could improve translatability of preclinical studies.

Recently, a new DMD mouse model was generated by crossing BL10-*mdx* mice on a DBA/2J [D2-wild type (WT)] genetic background (18). These D2.B10-*Dmd^mdx^*/J (D2-*mdx*) mice display a more severe dystrophic phenotype than BL10-*mdx* mice, including impaired muscle function and regeneration, decreased muscle weight, and elevated levels of fibrotic tissue in skeletal muscles (18, 19). As such, these mice may provide a promising alternative to the BL10-*mdx* model. Although the full genetic characterization of the D2-*mdx* strain underlying this more severe pathology is still pending, their polymorphism in *Ltbp4* was identified as a genetic modifier. Namely, a deletion in the coding region of the *Ltbp4* gene, which modifies activity of *Tgf*-β signaling was associated with increased SMAD signaling and fibrosis (20). Notably, the human *LTBP4* gene also contains polymorphisms that influence TGF-β activity, and indeed the *LTBP4* haplotype also affects loss of ambulation age of patients with DMD (21, 22). D2-*mdx* mice also have a dysfunctional *Anxa6* gene, which results in defects in the satellite cells’ self-renewal ability, and thus decreased muscle repair, in contrast to the BL10-*mdx* strain (23). Lastly, D2-*mdx* mice carry the *dyscalc1* gene locus, which is thought to be responsible for calcifications in their skeletal and heart muscles through the potential causative genes *Abcc6* or *Emp3* (24).

To date, there are only a few publications that investigate muscle pathology in the D2-*mdx* strain (18, 19, 23, 25, 26). A more comprehensive natural history study (*e*.*g.*, which includes longitudinal functional assessments), is needed to investigate the true potential of this emerging model. In fact, the “of mouse and measures” initiative was established in a collaborative effort of researchers working with the D2-*mdx* strain to compile data and align future efforts to establish natural history (27). We present our natural history data of the D2-*mdx* strain, generated using standardized operating procedures available from the TREAT-NeuroMuscular Disease (NMD) Alliance for the BL10-*mdx* strain. These data will be pivotal to generate generally accepted standardized outcome measures and protocols for the D2-*mdx* strain, which will facilitate high-quality preclinical studies and thereby enhance translatability to the clinic.

## MATERIALS AND METHODS

### Animals

Mice were housed under pathogen-free conditions in individually ventilated cages in rooms with a 12-h light/dark cycle at a temperature of 20.5°C and 40–60% humidity. *Ad libitum* access to standard RM3 chow (SDS, Essex, United Kingdom) and water was given to mice and they were handled according to the guidelines established by the Animal Experiment Committee (Dierexperimentencommissie) of the Leiden University Medical Center. All experiments were carried out under the approved protocol (13211). Efforts were made to minimize the burden and distress of the animals. D2-WT mice (Jax Stock 000671) were purchased from The Jackson Laboratory (Bar Harbor, ME, USA), whereas the other strains were acquired from in-house breeding.

### Experimental setup

At the age of 10 wk, C57BL/10ScSn-*Dmd^mdx^*/J (BL10-*mdx*) and D2.B10-*Dmd^mdx^*/J (D2-*mdx*) (both *n* = 6 males and *n* = 6 females) and C57BL/10ScSnJ (BL10-WT) and D2-WT (both *n* = 6 males) mice were euthanized by cervical dislocation to compare muscle pathology between the strains. The quadriceps, gastrocnemius, tibialis anterior, triceps, diaphragm, and heart were isolated, snap frozen in liquid nitrogen cooled isopentane, and used for histologic and gene analysis.

Another group of D2-*mdx* (*n* = 20, 10 males and 10 females) and D2-WT (*n* = 12, 6 males and 6 females) mice were subjected to a functional test regime. This functional test regime consisted of the forelimb grip strength test and 2 and 4 limb hanging tests. These tests were performed twice monthly from the age of 4–34 wk. Standardized operating procedures from the TREAT-NMD Alliance available for the BL10-*mdx* mouse model were used when possible (28). Body weights were recorded before every test session. At 34 wk of age, mice were euthanized by cervical dislocation, and muscles and heart were isolated. Gastrocnemius muscle and the heart were weighed.

### Forelimb grip strength test

Grip strength of the forelimbs was assessed using a grid attached to an isometric force transducer (Columbus Instruments, Columbus, OH, USA) according to the previously published protocol (28) and TREAT-NMD SOP DMD_M.2.2.001. The force transducer recorded the maximum force that was required to break the mouse’s grip from the mesh surface. In total 5 strength measurements, each containing of 3 pulls, were recorded. Three highest values were averaged and normalized to body weight.

### Two and 4 limbs hanging tests

The 2 and 4 limbs hanging tests were assessed according to the previously described protocols (28) and TREAT-NMD SOP DMD_M.2.1.004. For the 2 limbs hanging test, the mouse was suspended above a metal wire that was located 40 cm above a cage with soft beddings. After the mouse grasped the wire with its forelimbs, it was released, and the hanging time was recorded. For the 4 limbs hanging test, the mouse was placed on a grid, which was then turned upside down, 15 cm above a cage filled with soft bedding, after which hanging time was recorded. Both tests were completed after a hanging time of 600 s was achieved or after 3 sessions. The maximum hanging time was used for analysis.

### Respiratory function analysis

Respiratory function was measured with whole-body plethysmography (RM-80; Columbus Instruments) (29) at the age of 7, 14, and 34 wk. After 30 s of acclimatization period, the respiration signal was assessed for 120 s using a MiniDigi digitizer and Axoscope 10 software (Molecular Devices, San Jose, CA, USA). The data were analyzed with the event detection feature of the Clampfit 10 program (Molecular Devices). Using this monitoring system, respiration rate and amplitude were measured. The respiration amplitude was normalized to body weight.

### Creatine kinase level analysis

Creatine kinase (CK) levels were assessed on a weekly basis until the age of 8 wk and on a biweekly basis from 10 until 32 wk. To this end, blood was collected *via* a small angled cut in the tail in a heparin-coated Microvettes CB 300 (Sarstedt, Nümbrecht, Germany) and stored on ice for a maximum of 2 h. Subsequently, blood samples were centrifuged at 4°C for 5 min at 18,000 *g*. The obtained plasma was used to measure CK levels with Reflotron CK test strips in the Reflotron plus machine (Roche, Basel, Switzerland).

### Muscle histology

Freshly isolated muscles embedded in Optimal Cutting Temperature (OCT) compound (TissueTek; Sakura Finetek, Torrance, CA, USA), were snap frozen in 2-methylbutane (MilliporeSigma, Burlington, MA, USA), cooled in liquid nitrogen, and stored at −80°C until further processing. Sections of 8 μm were obtained with a Cryostat (CM3050 S Research Cryostat; Leica Microsystems, Buffalo Grove, IL, USA) for histologic analyses, whereas the remaining tissue was collected in 1.4 mm Zirconium Beads prefilled tubes (OPS Diagnostics, Lebanon, NJ, USA) and used for RNA isolation.

### Haematoxylin and eosin staining

Sections were fixed in ice-cold acetone for 5 min. Fixed tissue sections were stained with hematoxylin and eosin (H&E) (MilliporeSigma) according to the conventional protocol and imaged with BZ-X700 microscope (Keyence, Osaka, Japan) with a ×10 objective. The pictures, covering the entire cross-sectional area, were stitched using BZ-X700 analyzer software (Keyence). For removal of background noise, Adobe Photoshop CC 2014 (Adobe, San Jose, CA, USA) was used. Using this staining, fibrotic/necrotic/regenerated areas were quantified by calculating unhealthy/healthy ratio by ImageJ (National Institutes of Health, Bethesda, MD, USA) software using the color deconvolution plugin (*https://imagej.net/Deconvolution*) by 2–5 examiners, and the median of their assessments was used for analysis. Because the pathologic tissue was defined by the color deconvolution plugin by a lack of eosin staining, calcified fibers were not taken into consideration as these stain with eosin.

### Alizarin Red staining

To analyze calcification in muscle, sections were fixed in ice-cold acetone for 10 min and exposed to Alizarin Red staining solution (MilliporeSigma) for 1 min. Directly after, the sections were washed in acetone (Thermo Fisher Scientific, Waltham, MA, USA) for 30 s and in 1:1 acetone/xylene for 15 s. The sections were incubated in xylene for 1.5 h, mounted in Pertex and imaged with BZ-X700 microscope (Keyence) with a ×10 objective. The pictures were stitched using BZ-X700 analyzer software (Keyence). For background correction, Adobe (San Jose, CA, USA) Photoshop CC 2014 was used. The percentage of calcified tissue was determined by dividing Alizarin Red–positive areas by the total area using ImageJ software by 2–3 examiners, and the median of their assessments was used for analysis.

### Fiber-size measurement

For fiber-size analysis, muscle sections were blocked in PBS containing 0.05% Tween and 5% horse serum for 30 min at room temperature, stained with a laminin primary antibody (ab11575, dilution 1:100; Abcam, Cambridge, MA, USA) diluted in PBS/0.05%Tween at 4°C overnight. After washing with PBS, sections were incubated with secondary antibody goat-anti-rabbit IgG Alexa 594 (A11012, dilution 1:1000; Thermo Fisher Scientific) for 1 h at room temperature and mounted with Prolong gold mounting medium with DAPI (Thermo Fisher Scientific). Five microscopic views were analyzed with BZ-X700 analyzer (Keyence) with a ×10 objective, resulting in a mean total number of 1283–4487 fibers for quadriceps, 2072–4792 for gastrocnemius, 1473–3410 for triceps, and 2696–7213 for diaphragm measured, of which the number of fibers in a given fiber area class (500 μm^2^/class) was determined. The fibers with an area outside <100 µm^2^ or >10,000 µm^2^ were excluded from analysis.

### Embryonic myosin heavy chain staining

To detect regenerative fibers, sections were stained with a MYH3 primary antibody (F1.652, sc-53091 dilution 1:20; Santa Cruz Biotechnology, Dallas, TX, USA) overnight at 4°C and with a goat-anti-mouse Alexa 488 secondary antibody (A11001, dilution 1:1000; Thermo Fisher Scientific) for 1 h at room temperature and mounted with Prolong gold mounting medium with DAPI (Thermo Fisher Scientific). Images of the stained muscle sections were taken with a BZ-X700 fluorescent microscope (Keyence) with a ×10 objective. For background correction, Adobe Photoshop CC 2014 was used. The total number of embryonic myosin heavy chain (eMHC)-positive fibers was manually counted and divided by the total muscle area that was assessed using ImageJ software and reported as positive fibers per square millimeters.

### Sirius Red staining

To quantify the levels of collagen, sections were fixed in 4% paraformaldehyde for 10 min, followed by the fixation in 100% ethanol for 5 min and air dried for 30 min. The muscle sections were stained with Sirius Red solution (MilliporeSigma) for 45 min. Thereafter, sections were washed with 0.5% acetic acid water followed by rinsing with deionized water. The muscle sections underwent dehydration steps and were mounted in Pertex mounting medium (Histolab, Los Angeles, CA, USA). The stained muscle sections were imaged using a BZ-X700 fluorescent microscope (Keyence) with a ×10 objective and processed and stitched using BZ-X Analyzer (Keyence). The Sirius Red–positive areas were quantified using ImageJ and normalized to the total area. Two examiners assessed the images and the median of their results was used for analysis.

### Gene expression analysis

Muscle sections were collected in 1.4 mm Zirconium Beads prefilled tubes (OPS Diagnostics) and homogenized in TRIsure isolation reagent (Bioline, London, United Kingdom) using a MagNA Lyser (Roche). Total RNA was isolated using the TRIsure isolation method. The RNA was further purified (including DNase digestion step) by applying a NucleoSpin RNA II kit (Macherey-Nagel, Düren, Germany) according to the manufacturer’s instruction. From 300 to 500 ng of RNA (RNA input varied between muscles but was kept constant between groups), cDNA was synthesized using random N6 primers (Thermo Fisher Scientific) and Bioscript enzyme (GC Biotech, Waddinxveen, The Netherlands) according to the manufacturer’s instructions. Quantitative PCR (qPCR) was performed in triplo per biologic sample with the use of the LightCycler 480 and the ready-to-use SensiMix reagents (GC Biotech). The expression levels were analyzed applying the LinReg qPCR method (30) and normalized to the reference gene glyceraldehyde-3-phosphate dehydrogenase. Functional descriptions of genes analyses and their primer sequences are listed in Supplemental Table S1.

### Statistical analyses

Prism 4 (GraphPad Software, La Jolla, CA, USA) and SPSS 17.0.2 (IBM, White Plains, NY, USA) were used to analyze the data. A linear regression model was applied to compare the maximum hanging times of the hanging tests, grip strength, and CK levels *vs.* age per mouse per genotype. Respiration amplitudes and rates were compared between genotypes using mixed models.

Results of histologic and gene expression analyses were compared between genotypes using a 2-way ANOVA test and corrected for multiple comparisons with Tukey’s multiple comparison test. Fiber-size distribution was assessed using logistic regression with SPSS to demonstrate the switch toward smaller fiber sizes for dystrophic mice. Statistical significance was set at *P* < 0.05. Values are presented as means ± sd or ± sem, as indicated.

## RESULTS

### At a young age (10 wk), D2-*mdx* mice are more severely affected than BL10-*mdx* mice

**Figure 1:**
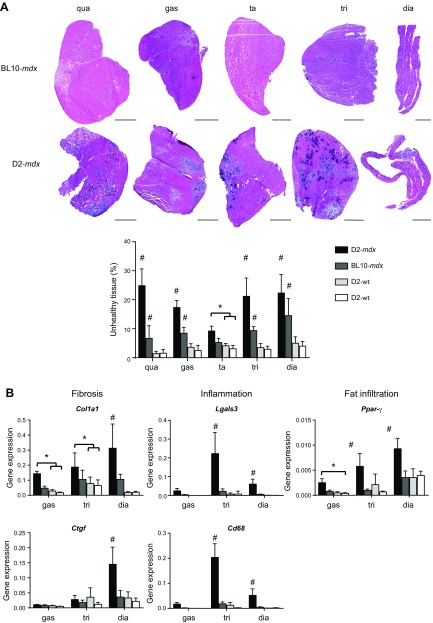
Histopathology of 10-wk-old mice. *A*) Representative H&E staining of 5 muscles belonging to 1 individual D2-*mdx* and BL10-*mdx* male. Scale bars, 1000 μm. Pathology consisted of necrosis, regenerated fibers, fibrosis, inflammation, and calcification and was more severe in D2-*mdx* mice. Differences between muscles were observed with the tibialis anterior being the least and diaphragm the most severely affected muscle in both dystrophic strains. *B*) Expression analysis of genes involved in fibrosis (*Col1a1*, *Ctgf*), inflammation (*Cd68*, *Lgals3*), and fat infiltration (*Pparγ*). Dia, diaphragm; gas, gastrocnemius; qua, quadriceps; ta, tibialis anterior; tri, triceps. Values represent means ± sd; *n* = 6 males/strain. ^#^Significant difference compared with all other genotypes; *P < 0.05.

To confirm and expand on the existing body of evidence that D2-*mdx* mice are more severely affected than BL10-*mdx* mice, we analyzed 6 D2-*mdx* and BL10-*mdx* males and females and 6 D2-WT and BL10-WT males at the age of 10 wk. Several dystrophic characteristics were compared between strains, genders, and different skeletal muscles. Overall histopathology of the quadriceps, gastrocnemius, tibialis anterior, triceps, and diaphragm was assessed with a H&E staining, quantifying the percentage of pathologic tissue (necrosis, regeneration, fibrosis, and inflammation). All male D2-*mdx* muscles analyzed were more severely affected than muscles from age- and gender-matched WT strains (**Fig. 1*A***). Moreover, histopathology of BL10-*mdx* mice was worse than that of both wild types for most muscles, except for the tibialis anterior. Overall pathology comprised >10% in D2-*mdx* quadriceps, gastrocnemius, triceps, and diaphragm but was significantly (*P* < 0.05) less severe in the tibialis anterior. It was evident that the gastrocnemius and diaphragm of both sexes were equally affected in both dystrophic strains (Supplemental Fig. S1*A*). Notably, pathology of the quadriceps of D2-*mdx* males was more severe (*P* < 0.05) than that of D2-*mdx* females. For the BL10-*mdx* strain, males and females were similarly affected.

To compare pathology in more depth between the strains and muscles, we assessed expression levels of genes involved in fibrosis (*Col1a1* and *Ctgf*), inflammation (*Lgals3* and *Cd68*) and fat infiltration (*Pparγ*) in males (Fig. 1*B*). Generally speaking, expression levels of these pathologic markers were higher in D2-*mdx* than in BL10-*mdx* mice and both wild-type strains, reaching significance for the majority of genes in the triceps and diaphragm. Expression levels of *Col1a1* and *Pparγ* were also significantly elevated in the gastrocnemius.

### D2-*mdx* muscles show heterotopic ossification

**Figure 2:**
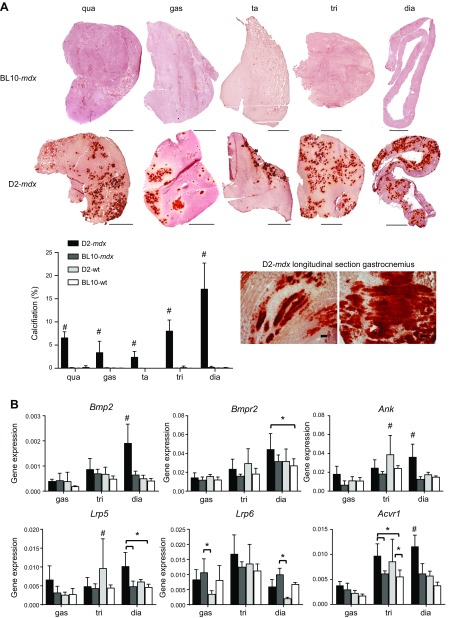
Myofiber calcification is extensive in 10-wk-old D2-*mdx* males. *A*) Representative Alizarin Red staining of 5 muscles belonging to the same D2-*mdx* and BL10-*mdx* males as shown in Fig. 1*A*. Scale bars, 1000 μm. Calcifications stain dark red and were primarily observed in D2-*mdx* mice. Longitudinal sections reveal that the calcified areas involve the entire myofiber and are contained within the fiber membrane. Quantifications revealed differences in severity between muscles. *B*) Expression analysis of genes involved in osteogenesis. Dia, diaphragm; gas, gastrocnemius; qua, quadriceps; ta, tibialis anterior; tri, triceps; *n* = 6 males/strain. Values represent means ± sd. ^#^Significant difference compared with all other genotypes; *P < 0.05.

**Figure 5:**
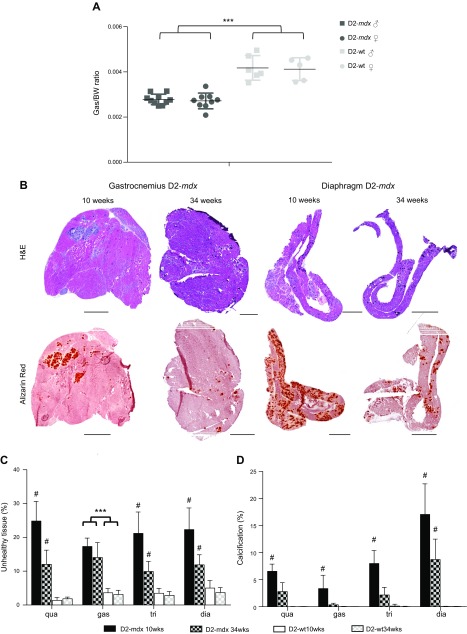
Histopathology is ameliorated in 34-wk-old D2-*mdx* mice. *A*) Gastrocnemius/body weight (GC/BW) ratio was smaller in D2-*mdx* mice. *B*) Representative H&E and Alizarin Red staining of the gastrocnemius and diaphragm belonging to a 10- and 34-wk-old D2-*mdx* male. The transient nature of the calcifications is clearly visible. Scale bars, 1000 μm. *C*, *D*) Overall pathology (*C*) and calcifications (*D*) were less pronounced in the older mice; *n* = 6 males/strain, except for 34-wk-old D2-*mdx* mice, which consisted of *n* = 10 males. Values represent means ± sd. ^#^Statistically significant difference compared with all other genotypes; ***P < 0.0001.

D2-WT mice carry a mutation in the *Dyscalc1* locus, which is involved in bone development and causative for a higher incidence of calcified lesions in muscles and testes (31, 32). Although heterotopic ossification has been previously observed in D2-*mdx* mice (19), the cause and extent to which myofibers devoid of dystrophin are susceptible to calcification is unknown. We therefore stained several skeletal muscles of 10-wk-old mice with Alizarin Red and performed gene expression pathway analyses. Myofiber calcification was observed occasionally in BL10-*mdx* and rarely in wild-type mice. However, D2-*mdx* mice had extensive calcification in all muscles (*P* < 0.05) (**Fig. 2*A***), which appeared to be of transient nature (**Fig. 5*B, D***). Interestingly, the calcifications coocurred often with degeneration (*e.g.*, necrotic and inflammatory regions). Longitudinal sections revealed that the calcifications occur within myofibers and often spread through the entire fiber. The abundance of calcified myofibers differed between muscles; the highest levels were observed in diaphragm (*P* < 0.0001), followed by triceps and quadriceps. Significantly lower amounts were observed in gastrocnemius and tibialis anterior (*P* < 0.01). Comparing genders, no significant differences were observed in both strains (Supplemental Fig. S1*B*). BL10-*mdx* muscles of both genders occasionally contained calcified fibers.

Little is known about the basis of the aberrant calcifications in skeletal muscle of D2-*mdx* mice. To determine whether osteogenesis is responsible, several bone differentiation genes [*Bmp2*, *Bmpr2*, *Ank*, *Lrp5*, *Lrp6*, and activin A receptor, type I (*Acvr1*)] were tested by qPCR. In line with histologic examination, the majority of genes were up-regulated, especially in the diaphragm of D2-*mdx* males and to a lesser extent in the gastrocnemius and triceps (Fig. 2*B*). Expression levels did not differ between BL10-*mdx* and their corresponding WT strains. Notably, in the triceps, expression levels of *Ank* and *Lrp5* in D2-WT mice exceeded that of BL10-*mdx* and BL10-WT mice. Results indicate that the observed calcification in muscle indeed could result from active osteogenesis.

### Muscle regeneration is altered in D2-*mdx* males

**Figure 3:**
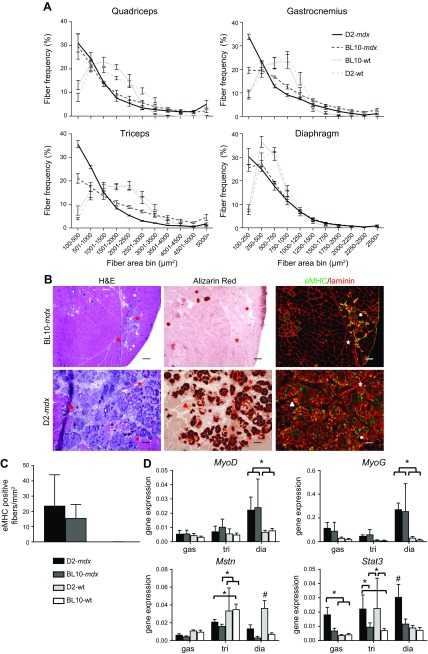
Regenerative capacity is not severely affected in 10-wk-old D2-*mdx* males. *A*) Fiber-size distributions were assessed for quadriceps, gastrocnemius, triceps, and diaphragm of the 4 strains. Overall, D2-*mdx* and BL10-*mdx* mice had significantly smaller fibers compared with WT strains. D2-*mdx* mice had a larger proportion of small fibers in gastrocnemius and triceps than BL10-*mdx* mice. *X*-axis labeling of quadriceps, triceps, and gastrocnemius are identical. *B*) Representative H&E, Alizarin Red, and eMHC stainings of consecutive sections of a D2-*mdx* and a BL10-*mdx* male quadriceps. Scale bars, 100 μm. Symbols indicate corresponding fibers. eMHC-positive fibers colocalized to areas of inflammation and calcification. *C*) eMHC-positive fibers were manually counted on an entire cross section. Abundance did not differ between D2-*mdx* and BL10-*mdx* males. *D*) Expression analysis of genes involved in regeneration. Dia, diaphragm; gas, gastrocnemius; tri, triceps; *n* = 6 males/strain. Values represent means ± sem (*A*) and mean ± sd (*B*–*D*). ^#^Significant difference compared with all other genotypes; *P < 0.05.

Fiber-size distributions were assessed for quadriceps, gastrocnemius, triceps, and diaphragm of the 4 strains. Overall, D2-*mdx* and BL10-*mdx* mice had much more small sized fibers (<1000 μm^2^) than WT mice (**Fig. 3*A***). For the quadriceps and diaphragm, no differences were observed in fiber-size distribution between the dystrophic strains, whereas in the gastrocnemius and triceps, the proportion of small fibers in D2-*mdx* mice exceeded that of BL10-*mdx* mice (gastrocnemius; 33.8 *vs.* 19.6% triceps; 35.6 *vs.* 21.1% for D2-*mdx* and BL10-*mdx* mice, respectively). In both muscles, the proportion of fibers with an area of >2000 µm^2^ was smaller in D2-*mdx* than in BL10-*mdx* mice.

To assess the regenerative capacity of D2-*mdx* mice, we stained the quadriceps for eMHC-positive fibers and assessed their abundance in whole-muscle cross-sections per square millimeters (Fig. 3*B, C*). Whereas no eMHC-positive fibers were observed in the wild-type strains, both D2-*mdx* and BL10-*mdx* mice, respectively, had 23.7 and 12.5 eMHC-positive myofibers per square millimeters. In D2-*mdx* mice, eMHC-positive fibers were predominantly found in calcified areas (Fig. 3*B, C*). We also assessed expression of genes involved in regeneration (*MyoD*, *MyoG*, *Mstn*, and *Stat3*) (Fig. 3*D*). *MyoD* and *MyoG* levels in diaphragm were elevated to similar extend in both dystrophic strains compared with WT but not in gastrocnemius or triceps. *Mstn* levels in the diaphragm were reduced in D2-*mdx* compared with D2-WT mice. *Stat3* expression was up-regulated in D2-*mdx* gastrocnemius and diaphragm, but not in the triceps, compared D2-WT mice.

### Muscle performance is severely affected in D2-*mdx* males and deteriorates with age

**Figure 4:**
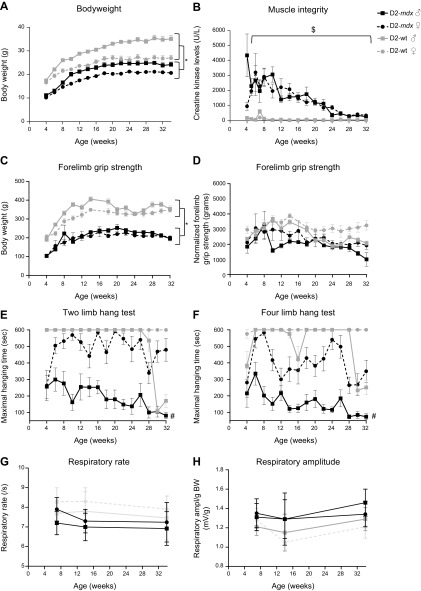
A gender and age comparison for D2-*mdx* and D2-WT mice shows that muscle function is severely affected in D2-*mdx* males, but not females, and deteriorates with age. *A*) D2-*mdx* mice were lighter than wild types. *B*) CK levels were elevated in young D2-*mdx* mice and dropped with age. *C, D*) Forelimb grip strength (*C*) was impaired in D2-*mdx* mice, but when normalized to body weight (*D*), this difference lost its significance. *E*, *F*) Performance in the 2 limb hanging test (*E*) and 4 limb hanging test (*F*) was severely impaired in D2-*mdx* males but not in females. *G*) Respiratory rate was decreased in D2-*mdx* mice, although this did not reach significance. *H*) Respiratory amplitude normalized to body weight showed a nonsignificant increase in D2-*mdx* mice. For *G* and *H*, significance was reached when data of both genders were pooled; *n* = 10 D2-*mdx* mice and *n* = 6 D2-WT mice of both genders. Values represent means ± sem. ^$^Significant difference compared with all other groups; significant drop with age in D2-mdx mice; *P < 0.05.

To longitudinally assess muscle function, we subjected 10 D2-*mdx* and 6 D2-WT mice of both genders to a biweekly functional test regime consisting of forelimb grip strength and 2 hanging tests starting at 4 wk of age. Weight measurements recorded over time confirmed the lower body weight in D2-*mdx* mice (**Fig. 4*A***). In both strains, males were heavier than females, although this difference was more pronounced in D2-WT mice. CK levels were assessed as a marker for myofiber integrity (Fig. 4*B*). CK levels were elevated in young D2-*mdx* mice regardless of their gender but decreased with age. From the age of 24 (males) or 26 (females) wk onwards, levels were below 500 U/L, which are considered as WT levels. Forelimb grip strength was significantly impaired in D2-*mdx* mice (*P* < 0.0001) compared with WT but did not differ between genders (Fig. 4*C*). Although the same pattern was observed when grip strength values were normalized to body weight, differences lost their statistical significance (Fig. 4*D*). Hanging performance was assessed with 2 hanging tests, using a wire with a starting position of 2 limbs (Fig. 4*E*) or a grid with a starting position of 4 limbs (Fig. 4*F*). Performance of D2-*mdx* males was significantly (*P* < 0.0001) impaired and deteriorated with age in both hanging tests as compared with WT mice. Notably, D2-*mdx* females outperformed males in both tests (2 limbs hang test *P* < 0.0001, 4 limbs hang test *P* < 0.0001). Performance of WT males, but not females, unexpectedly dropped in the last 2 wk for which we do not have an explanation. Respiratory rate and amplitude were assessed at 7, 14, and 34 wk of age (Fig 4*G, H*). Although D2-*mdx* mice of both genders consistently had a lower respiratory rate and higher amplitude than D2-WT mice, this reached statistical significance only when data of both genders were combined.

### Skeletal muscle pathology stabilizes in 34-wk-old D2-*mdx* males

At age 34 wk, the muscle/body weight ratio of the gastrocnemius was significantly lower in D2-*mdx* compared with wild-type mice of both genders (Fig. 5*A*). Muscle histopathology was assessed and compared with that of the 10-wk-old males. Because females outperformed males in the functional analyses, making females less suitable for future preclinical studies using muscle functionality as outcome measure, we excluded them from further skeletal muscle analyses. We also excluded the tibialis anterior from further analyses being the least severely affected muscle in 10-wk-old mice. Sections were H&E stained to assess the percentage of unhealthy tissue (necrosis, regeneration, fibrosis, and inflammation) (Fig. 5*B*). For all muscles, we observed a reduction in pathologic severity in the 34-wk-old D2-*mdx* males compared with the younger mice (Fig. 5*C*). However, at 34 wk, levels still exceeded that of WT with >10% of the muscle showing pathologic damage. Histopathology did not largely differ between the muscles. Similar to overall pathology, the extent of calcification was statistically significantly ameliorated in the muscles derived from the 34-wk-old D2-*mdx* mice (Fig. 5*B, D*). Diaphragm was the most affected, whereas only minor amounts of calcification were detected for the other skeletal muscles.

**Figure 6:**
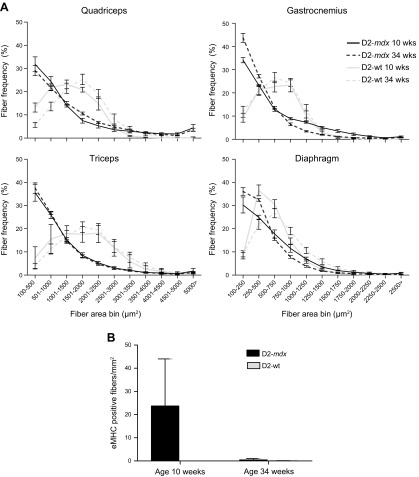
Fiber-size and regeneration in 34-wk-old D2-*mdx* mice. *A*) Fiber-size distributions were similar between younger and older D2-*mdx* mice for quadriceps and triceps. *X*-axis labeling of quadriceps, triceps, and gastrocnemius are identical. *B*) eMHC-positive fibers were only occasionally observed in old D2-*mdx* mice. Values represent means ± sd.

Furthermore, we analyzed fiber-size distributions in 34-wk-old mice and compared outcomes with those observed in 10-wk-old gender-matched mice. Similar to younger mice, large proportions of smaller fibers were observed in quadriceps and triceps for the older D2-*mdx* mice, and their abundance did not alter with age (**Fig. 6*A***). In the gastrocnemius and diaphragm, however, the smallest bin contained slightly more fibers in the older mice, which consequently had fewer fibers between 1000 and 1500 μm^2^.

We also assessed the amount of eMHC-positive fibers in the gastrocnemius and observed that at 34 wk of age there were few eMHC-positive fibers present, indicating that at this age active regeneration is not occurring (Fig. 6*B*).

### Cardiomyopathy in D2-*mdx* mice

**Figure 7:**
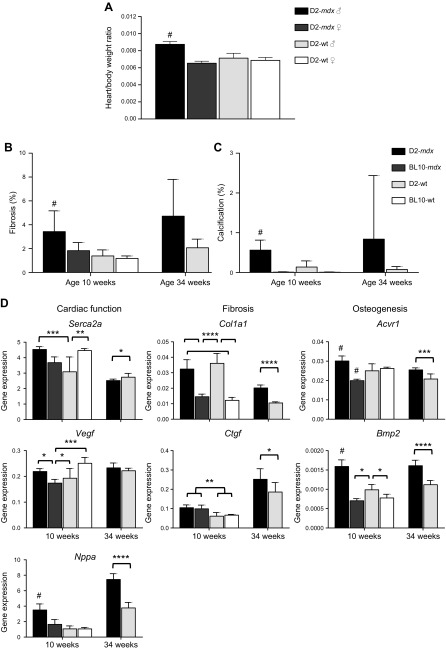
Pathology of the heart of D2-*mdx* and BL10-*mdx* mice at 10 and 34 wk of age. *A*) D2-*mdx* males, but not females, suffered from cardiac hypertrophy at the age of 34 wk. *B*) Despite large individual variation, D2-*mdx* mice had marginally more fibrotic lesions from 10 wk of age onwards compared with BL10-*mdx* mice. *C*) In contrast to BL10-*mdx* mice, D2-*mdx* mice had calcifications in the heart at 10 wk of age. *D*) Expression analysis of genes involved in cardiac function (*Serca2a*, *Vegf*, and *Nppa*), fibrosis (*Col1a1*, *Ctgf*) and osteogenesis (*Acvr1*, *Bmp2*); *n* = 6 males/strain, except for 34-wk-old D2-*mdx* mice, which consisted of *n* = 10 males. Values represent means ± sd. ^#^Statistically significant difference compared with all other genotypes; *P < 0.05, **P < 0.01, ***P < 0.001, ****P < 0.0001.

In contrast to BL10-*mdx* mice that develop heart pathology from ∼6 mo onwards, the D2-WT genetic background is known to develop cardiac calcifications at an early age (19). To confirm that dystrophin deficiency renders the heart more susceptible to damage in the DBA2/J genetic background, we assessed hypertrophy of the heart in 34-wk-old mice. The heart/body weight ratio in D2-*mdx* males was significantly larger (*P* < 0.05) compared with D2-*mdx* females and WT mice (**Fig. 7*A***).

The amount of fibrosis in the heart was assessed on sections stained with Sirius Red in D2-*mdx* and wild-type mice aged 10 and 34 wk (Fig. 7*B*). At 10 wk of age, D2-*mdx* mice already had significant fibrotic lesions. The extent appeared to increase with age in 34-wk-old D2-*mdx* mice, although mean levels did not significantly differ. Notably, at both ages, individual variation between mice was large, with some mice showing almost no fibrosis, whereas fibrosis was pronounced in others. We also assessed cardiac calcifications as D2-WT mice are known for developing these in the heart (Fig. 7*C*). We observed a few calcifications in both young and older D2-WT mice, which were not seen in BL10-WT mice. In line with our observations for fibrosis, also the extent of the calcifications varied in individual D2-*mdx* mice, ranging from a few fibers to extensive areas in 1 D2-*mdx* mouse. Such variation was present in both ages, although it was more pronounced in the older mice (varying between 1.3 and 5.9% at 10 wk, and 2.1–13.2% at 34 wk).

Expression analysis of genes involved in cardiac function, fibrosis, and osteogenesis were assessed by qPCR (Fig. 7*D*). Notably, already at 10 wk of age, expression levels of *Serca2a*, *Nppa*, *Ctgf*, *Acvr1*, and *Bmp2* were significantly higher in D2-*mdx* than in D2-WT mice. Expression levels of these genes did not differ that drastically between *mdx* and WT mice on a BL10 genetic background because only expression of *Vegf* and *Acvr1* was decreased, whereas that of *Ctgf* was increased in BL10-*mdx* mice. Heart pathology deteriorated with age at 34 wk, and for all genes except *Vegf*, a statistically significant difference was observed between the D2-*mdx* and D2-WT mice.

## DISCUSSION

Despite the potential of the D2-*mdx* model for preclinical research, to date, its natural history has not been thoroughly investigated, whereas the number of preclinical studies using this model is exponentially growing. Lacking natural history data, these intervention studies are at risk of being not well designed due to lack of knowledge on which age, gender, muscles, and outcome measures to select.

In this study, we examined the natural history of D2-*mdx* mice in detail by directly comparing the pathology of 5 different muscles with that of muscles from BL10-*mdx* mice at ages 10 and 34 wk. Histopathology of 10-wk-old D2-*mdx* mice consisted of extensive necrosis, inflammation, fibrosis, and, notably, calcifications. Overall severity of these pathologic hallmarks was much worse compared with BL10-*mdx* mice. Accordingly, the majority of genes involved in these processes were, especially in the diaphragm, up-regulated to a larger extent in the D2-*mdx* mice. Differences in severity between the different muscles within mice matched between the 2 *mdx* strains (*i.e.*, diaphragm most severely and tibialis least severely affected). The exacerbated pathology likely results from the dysfunctional *Anax6* gene, which hampers membrane repair after injury especially in dystrophic muscle (33). It thereby further triggers necrosis and chronic inflammatory responses evident by observed elevated *Lgals3* and *Cd68* expression. In line, increased inflammatory cytokines and Evans blue dye staining has been observed in young D2-*mdx* mice (19). The increased levels of fibrosis (*i.e.*, elevated *Col1a1* expression) are likely caused by the dysfunctional *Ltbp4* gene, which increases TGF-β signaling (20). LTBP4 is a known genetic modifier in patients with DMD (34). The majority of muscles showed an increase in the number of small fibers in D2-*mdx* compared with BL10-*mdx* mice. With age, the amount of small fibers even further increased. We assessed expression of several genes involved in regeneration and stained for eMHC-positive fibers to assess regenerative capacity. Especially *Stat3* levels were significantly up-regulated in D2-*mdx* mice, whereas no difference in eMHC expression was observed. Also, Coley *et al.* (19) described comparable levels of regenerative gene markers and BrdU uptake at 6 wk of age. However, they also observed a reduction in centralize nucleated fibers. Others confirmed the low centralized nuclei counts and described reductions in revertant fibers, all indicative for an impaired regenerative capacity (18, 23). With studies lacking on the onset and extent of necrosis in the D2-*mdx* model, it is too early to establish whether differences are caused by a reduction in necrosis or a reduction in regeneration in the D2-*mdx* model. Further research on the extent of necrosis and the regenerative capacity of the D2-*mdx* model in younger mice is needed.

One striking characteristic of the D2-*mdx* strain is the presence of high numbers of calcified fibers. Although occasional calcification was observed in BL10-*mdx* mice, all examined D2-*mdx* muscles contained extensive groups of calcified fibers that were restricted to areas of muscle necrosis and inflammation. This colocalization could implicate a direct causative link between these pathologic events. We show that several genes within the same bone differentiation pathway are up-regulated in D2-*mdx* mice. We believe that activation of *Bmp2* *via* increased inflammatory cytokine levels initiates this cascade, eventually triggering *Ank* expression and subsequently osteogenesis. Whether the pronounced calcifications catalyze myonecrosis or impair regeneration and thereby further exacerbate disease pathology is unknown and should be further studied. Furthermore, we observed that the calcifications are of transient nature because their abundance significantly reduced in 34-wk-old D2-*mdx* mice. In addition, other features of disease pathology significantly ameliorated with age in D2-*mdx* mice as well, which is in line with the disease stabilization observed in BL10-*mdx* mice. This again implicates a direct link between the occurrence of calcifications and myonecrosis/inflammation, where the calcifications resolve in absence of active necrosis/regeneration. The reason why myofiber calcification results from processes involved in necrosis and regeneration in growing young mice and why it has a transient nature is unknown and should be further investigated. Lastly, we focused on body weight and muscle function and their applicability for the D2-*mdx* model and performed longitudinal functional assessments in both genders. Like others, we observed that D2-*mdx* muscles are atrophic, which is the opposite of the hypertrophy associated with the BL10-*mdx* strain. This atrophic phenotype is more comparable to the pseudohypertrophy found in patients with DMD due to extensive fibrosis and fat infiltrations.

Interestingly, in the hanging tests, D2-*mdx* females outperformed males. Also, for the BL10-*mdx* mouse (35, 36) and SGCD-null mice for LGMD2F (37) it has been reported that females exhibit less severe deficits in muscle function than males. In contrast, in the SGCA-null mouse for LGMD2D, no difference in functionality between genders has been observed (37). The underlying mechanisms of gender differences in some muscular dystrophies are still largely inconclusive. Most likely there is a hormonal effect. Interestingly, treatment with tamoxifen, an estrogen analog, was found to have beneficial effects on BL10-*mdx* mice, and the therapeutic effect of tamoxifen treatment is currently being explored (*http://www.clinicaltrials.gov**;* identifier: NCT03354039) in a clinical trial in patients with DMD.

Importantly, however, these observations highlight the need of a single gender (preferably males) in preclinical studies using D2-*mdx* mice. In line with previous observations in BL10-*mdx* mice (29, 38), respiratory function at rest was not severely impaired in D2-*mdx* mice, a changed respiratory capacity as compared with WT was only statistically significant when data of both genders were combined. Although we hypothesized that the more severely diaphragm of D2-*mdx* mice would more significantly affect respiratory function, like in *mdx-utrn^−/−^* mice (29), this was not observed. Further *ex vivo* experiments into the physiology of the diaphragm are warranted to further investigate this.

In D2-*mdx* mice, heart pathology is evident at an earlier age than in BL10-*mdx* mice. Already at 10 wk of age, some occasional cardiac calcifications are present in D2-*mdx* mice, which increase in severity with age. Notably, also in D2-WT mice, cardiac calcifications are observed, but these are exacerbated due to the dystrophin absence in D2-*mdx* mice. In agreement, others have shown that heart function becomes impaired already at a relatively young age in D2-*mdx* mice (19).

Taken together, the natural history study presented underlines the preferential use of male mice in preclinical studies where muscle function is one of the key outcome measures. Furthermore, it indicates that histopathology (especially calcifications) are severe in D2-*mdx* mice and that this ameliorates with age. Future studies are required to study the timing of this amelioration, necrosis, and regeneration in more detail, as also outlined in the report of the “of mouse and measures” initiative (27). Based on our natural history study, preclinical studies using this model should start interventions at early age, assessing therapeutic effects before the pathology has already ameliorated too much spontaneously.

## Supplementary Material

This article includes supplemental data. Please visit *http://www.fasebj.org* to obtain this information.

## Abbreviations

Acvr1: activin A receptor, type I
BL10-mdx: C57BL/10ScSn-*Dmd^mdx^*/J
BL10-WT: C57BL/10ScSnJ
CK: creatine kinase
D2-WT: DBA/2J
DMD: Duchenne muscular dystrophy
eMHC: embryonic myosin heavy chain
H&E: hematoxylin and eosin
LGMD: limb girdle muscular dystrophy
qPCR: quantitative PCR
WT: wild type

## References

[1] Mendell J. R., Rodino-Klapac L. R., Sahenk Z., Roush K., Bird L., Lowes L. P., Alfano L., Gomez A. M., Lewis S., Kota J., Malik V., Shontz K., Walker C. M., Flanigan K. M., Corridore M., Kean J. R., Allen H. D., Shilling C., Melia K. R., Sazani P., Saoud J. B., Kaye E. M.; Eteplirsen Study Group (2013) Eteplirsen for the treatment of Duchenne muscular dystrophy. Ann. Neurol. 74, 637–647 23907995 10.1002/ana.23982

[2] Moat S. J., Bradley D. M., Salmon R., Clarke A., Hartley L. (2013) Newborn bloodspot screening for Duchenne muscular dystrophy: 21 years experience in Wales (UK). Eur. J. Hum. Genet. 21, 1049–1053 23340516 10.1038/ejhg.2012.301 PMC3778339

[3] Straub V., Balabanov P., Bushby K., Ensini M., Goemans N., De Luca A., Pereda A., Hemmings R., Campion G., Kaye E., Arechavala-Gomeza V., Goyenvalle A., Niks E., Veldhuizen O., Furlong P., Stoyanova-Beninska V., Wood M. J., Johnson A., Mercuri E., Muntoni F., Sepodes B., Haas M., Vroom E., Aartsma-Rus A. (2016) Stakeholder cooperation to overcome challenges in orphan medicine development: the example of Duchenne muscular dystrophy. Lancet Neurol. 15, 882–890 27302365 10.1016/S1474-4422(16)30035-7

[4] Emery A. E. (2002) The muscular dystrophies. Lancet 359, 687–695 11879882 10.1016/S0140-6736(02)07815-7

[5] Kim S., Campbell K. A., Fox D. J., Matthews D. J., Valdez R.; MD STARnet (2015) Corticosteroid treatments in males with Duchenne muscular dystrophy: treatment duration and time to loss of ambulation. J. Child Neurol. 30, 1275–1280 25414237 10.1177/0883073814558120 PMC4439376

[6] Moxley R. T. III, Pandya S., Ciafaloni E., Fox D. J., Campbell K. (2010) Change in natural history of Duchenne muscular dystrophy with long-term corticosteroid treatment: implications for management. J. Child Neurol. 25, 1116–1129 20581335 10.1177/0883073810371004

[7] Emery A. E. H., Muntoni F., Quinlivan R. C. M. (2015) Duchenne Muscular Dystrophy, Oxford University Press, Oxford, United Kingdom

[8] Bladen C. L., Salgado D., Monges S., Foncuberta M. E., Kekou K., Kosma K., Dawkins H., Lamont L., Roy A. J., Chamova T., Guergueltcheva V., Chan S., Korngut L., Campbell C., Dai Y., Wang J., Barišić N., Brabec P., Lahdetie J., Walter M. C., Schreiber-Katz O., Karcagi V., Garami M., Viswanathan V., Bayat F., Buccella F., Kimura E., Koeks Z., van den Bergen J. C., Rodrigues M., Roxburgh R., Lusakowska A., Kostera-Pruszczyk A., Zimowski J., Santos R., Neagu E., Artemieva S., Rasic V. M., Vojinovic D., Posada M., Bloetzer C., Jeannet P. Y., Joncourt F., Díaz-Manera J., Gallardo E., Karaduman A. A., Topaloğlu H., El Sherif R., Stringer A., Shatillo A. V., Martin A. S., Peay H. L., Bellgard M. I., Kirschner J., Flanigan K. M., Straub V., Bushby K., Verschuuren J., Aartsma-Rus A., Béroud C., Lochmüller H. (2015) The TREAT-NMD DMD Global Database: analysis of more than 7,000 Duchenne muscular dystrophy mutations. Hum. Mutat. 36, 395–402 25604253 10.1002/humu.22758 PMC4405042

[9] Hoffman E. P., Brown R. H. Jr., Kunkel L. M. (1987) Dystrophin: the protein product of the Duchenne muscular dystrophy locus. Cell 51, 919–928 3319190 10.1016/0092-8674(87)90579-4

[10] Gissel H. (2005) The role of Ca2+ in muscle cell damage. Ann. N. Y. Acad. Sci. 1066, 166–180 16533926 10.1196/annals.1363.013

[11] Klingler W., Jurkat-Rott K., Lehmann-Horn F., Schleip R. (2012) The role of fibrosis in Duchenne muscular dystrophy. Acta Myol. 31, 184–195 23620650 PMC3631802

[12] Wallace G. Q., McNally E. M. (2009) Mechanisms of muscle degeneration, regeneration, and repair in the muscular dystrophies. Annu. Rev. Physiol. 71, 37–57 18808326 10.1146/annurev.physiol.010908.163216

[13] Sicinski P., Geng Y., Ryder-Cook A. S., Barnard E. A., Darlison M. G., Barnard P. J. (1989) The molecular basis of muscular dystrophy in the mdx mouse: a point mutation. Science 244, 1578–1580 2662404 10.1126/science.2662404

[14] DiMario J. X., Uzman A., Strohman R. C. (1991) Fiber regeneration is not persistent in dystrophic (MDX) mouse skeletal muscle. Dev. Biol. 148, 314–321 1936568 10.1016/0012-1606(91)90340-9

[15] Boldrin L., Zammit P. S., Morgan J. E. (2015) Satellite cells from dystrophic muscle retain regenerative capacity. Stem Cell Res. (Amst.) 14, 20–29 10.1016/j.scr.2014.10.007 PMC4305370 25460248

[16] Grounds M. D., Radley H. G., Lynch G. S., Nagaraju K., De Luca A. (2008) Towards developing standard operating procedures for pre-clinical testing in the mdx mouse model of Duchenne muscular dystrophy. Neurobiol. Dis. 31, 1–19 18499465 10.1016/j.nbd.2008.03.008 PMC2518169

[17] De Luca A. (2012) Pre-clinical drug tests in the mdx mouse as a model of dystrophinopathies: an overview. Acta Myol. 31, 40–47 22655516 PMC3440805

[18] Fukada S., Morikawa D., Yamamoto Y., Yoshida T., Sumie N., Yamaguchi M., Ito T., Miyagoe-Suzuki Y., Takeda S., Tsujikawa K., Yamamoto H. (2010) Genetic background affects properties of satellite cells and mdx phenotypes. Am. J. Pathol. 176, 2414–2424 20304955 10.2353/ajpath.2010.090887 PMC2861106

[19] Coley W. D., Bogdanik L., Vila M. C., Yu Q., Van Der Meulen J. H., Rayavarapu S., Novak J. S., Nearing M., Quinn J. L., Saunders A., Dolan C., Andrews W., Lammert C., Austin A., Partridge T. A., Cox G. A., Lutz C., Nagaraju K. (2016) Effect of genetic background on the dystrophic phenotype in mdx mice. Hum. Mol. Genet. 25, 130–145 26566673 10.1093/hmg/ddv460 PMC4690497

[20] Heydemann A., Ceco E., Lim J. E., Hadhazy M., Ryder P., Moran J. L., Beier D. R., Palmer A. A., McNally E. M. (2009) Latent TGF-beta-binding protein 4 modifies muscular dystrophy in mice. J. Clin. Invest. 119, 3703–3712 19884661 10.1172/JCI39845 PMC2786802

[21] Flanigan K. M., Ceco E., Lamar K. M., Kaminoh Y., Dunn D. M., Mendell J. R., King W. M., Pestronk A., Florence J. M., Mathews K. D., Finkel R. S., Swoboda K. J., Gappmaier E., Howard M. T., Day J. W., McDonald C., McNally E. M., Weiss R. B.; United Dystrophinopathy Project (2013) LTBP4 genotype predicts age of ambulatory loss in Duchenne muscular dystrophy. Ann. Neurol. 73, 481–488 23440719 10.1002/ana.23819 PMC4106425

[22] Van den Bergen J. C., Hiller M., Böhringer S., Vijfhuizen L., Ginjaar H. B., Chaouch A., Bushby K., Straub V., Scoto M., Cirak S., Humbertclaude V., Claustres M., Scotton C., Passarelli C., Lochmüller H., Muntoni F., Tuffery-Giraud S., Ferlini A., Aartsma-Rus A. M., Verschuuren J. J., ’t Hoen P. A., Spitali P. (2015) Validation of genetic modifiers for Duchenne muscular dystrophy: a multicentre study assessing SPP1 and LTBP4 variants. J. Neurol. Neurosurg. Psychiatry 86, 1060–1065 25476005 10.1136/jnnp-2014-308409 PMC4602257

[23] Rodrigues M., Echigoya Y., Maruyama R., Lim K. R., Fukada S. I., Yokota T. (2016) Impaired regenerative capacity and lower revertant fibre expansion in dystrophin-deficient mdx muscles on DBA/2 background. Sci. Rep. 6, 38371 27924830 10.1038/srep38371 PMC5141435

[24] Roberts N. W., Holley-Cuthrell J., Gonzalez-Vega M., Mull A. J., Heydemann A. (2015) Biochemical and functional comparisons of mdx and Sgcg(-/-) muscular dystrophy mouse models. BioMed Res. Int. 2015, 131436 26064876 10.1155/2015/131436 PMC4433636

[25] Vohra R., Batra A., Forbes S. C., Vandenborne K., Walter G. A. (2017) Magnetic resonance monitoring of disease progression in mdx mice on different genetic backgrounds. Am. J. Pathol. 187, 2060–2070 28826559 10.1016/j.ajpath.2017.05.010 PMC5809503

[26] Hakim C. H., Wasala N. B., Pan X., Kodippili K., Yue Y., Zhang K., Yao G., Haffner B., Duan S. X., Ramos J., Schneider J. S., Yang N. N., Chamberlain J. S., Duan D. (2017) A five-repeat micro-dystrophin gene ameliorated dystrophic phenotype in the severe DBA/2J-mdx model of Duchenne muscular dystrophy. Mol. Ther. Methods Clin. Dev. 6, 216–230 28932757 10.1016/j.omtm.2017.06.006 PMC5596503

[27] Gordish-Dressman H., Willmann R., Dalle Pazze L., Kreibich A., van Putten M., Heydemann A., Bogdanik L., Lutz C., Davies K., Demonbruen A. R., Duan D., Elsey D., Fukada S. I., Girgenrath M., Patrick Gonzalez J., Grounds M. D., Nichols A., Partridge T., Passini M., Sanarica F., Schnell F. J., Wells D. J., Yokota T., Young C. S., Zhong Z., Spurney C., Spencer M., De Luca A., Nagaraju K., Aartsma-Rus A. (2018) “Of mice and measures”: a Project to improve how we advance Duchenne muscular dystrophy therapies to the clinic. J. Neuromuscul. Dis. 5, 407–417 30198876 10.3233/JND-180324 PMC6218134

[28] Aartsma-Rus A., van Putten M. (2014) Assessing functional performance in the mdx mouse model. J. Vis. Exp. 10.3791/51303 PMC4158772 24747372

[29] Van der Pijl E. M., van Putten M., Niks E. H., Verschuuren J. J., Aartsma-Rus A., Plomp J. J. (2016) Characterization of neuromuscular synapse function abnormalities in multiple Duchenne muscular dystrophy mouse models. Eur. J. Neurosci. 43, 1623–1635 27037492 10.1111/ejn.13249

[30] Ruijter J. M., Ramakers C., Hoogaars W. M., Karlen Y., Bakker O., van den Hoff M. J., Moorman A. F. (2009) Amplification efficiency: linking baseline and bias in the analysis of quantitative PCR data. Nucleic Acids Res. 37, e45 19237396 10.1093/nar/gkp045 PMC2665230

[31] Aherrahrou Z., Doehring L. C., Kaczmarek P. M., Liptau H., Ehlers E. M., Pomarino A., Wrobel S., Götz A., Mayer B., Erdmann J., Schunkert H. (2007) Ultrafine mapping of Dyscalc1 to an 80-kb chromosomal segment on chromosome 7 in mice susceptible for dystrophic calcification. Physiol. Genomics 28, 203–212 16926270 10.1152/physiolgenomics.00133.2006

[32] Meng H., Vera I., Che N., Wang X., Wang S. S., Ingram-Drake L., Schadt E. E., Drake T. A., Lusis A. J. (2007) Identification of Abcc6 as the major causal gene for dystrophic cardiac calcification in mice through integrative genomics. Proc. Natl. Acad. Sci. USA 104, 4530–4535 17360558 10.1073/pnas.0607620104 PMC1838635

[33] Swaggart K. A., Demonbreun A. R., Vo A. H., Swanson K. E., Kim E. Y., Fahrenbach J. P., Holley-Cuthrell J., Eskin A., Chen Z., Squire K., Heydemann A., Palmer A. A., Nelson S. F., McNally E. M. (2014) Annexin A6 modifies muscular dystrophy by mediating sarcolemmal repair. Proc. Natl. Acad. Sci. USA 111, 6004–6009 24717843 10.1073/pnas.1324242111 PMC4000833

[34] Weiss R. B., Vieland V. J., Dunn D. M., Kaminoh Y., Flanigan K. M.; United Dystrophinopathy Project (2018) Long-range genomic regulators of THBS1 and LTBP4 modify disease severity in duchenne muscular dystrophy. Ann. Neurol. 84, 234–245 30014611 10.1002/ana.25283 PMC6168392

[35] Hourdé C., Joanne P., Noirez P., Agbulut O., Butler-Browne G., Ferry A. (2013) Protective effect of female gender-related factors on muscle force-generating capacity and fragility in the dystrophic mdx mouse. Muscle Nerve 48, 68–75 23625771 10.1002/mus.23700

[36] Hakim C. H., Duan D. (2012) Gender differences in contractile and passive properties of mdx extensor digitorum longus muscle. Muscle Nerve 45, 250–256 22246882 10.1002/mus.22275 PMC3298688

[37] Pasteuning-Vuhman S., Putker K., Tanganyika-de Winter C. L., Boertje-van der Meulen J. W., van Vliet L., Overzier M., Plomp J. J., Aartsma-Rus A., van Putten M. (2017) Natural disease history of mouse models for limb girdle muscular dystrophy types 2D and 2F. PLoS One 12, e0182704 28797108 10.1371/journal.pone.0182704 PMC5552258

[38] Van der Pijl E. M., van Putten M., Niks E. H., Verschuuren J. J. G. M., Aartsma-Rus A., Plomp J. J. (2018) Low dystrophin levels are insufficient to normalize the neuromuscular synaptic abnormalities of mdx mice. Neuromuscul. Disord. 28, 427–442 29631954 10.1016/j.nmd.2018.02.013

