# Supplementary material for: Natural disease history of the D2-mdx mouse model for Duchenne muscular dystrophy
Source: FASEB J. 2019 Apr 1;33(7):8110–24. doi: 10.1096/fj.201802488R (PMC6593893; doi:10.1096/fj.201802488R)
Supplement: Supplementary file 1 [file fj.201802488R.sf1.pdf]

Supplementary Figure 1

A

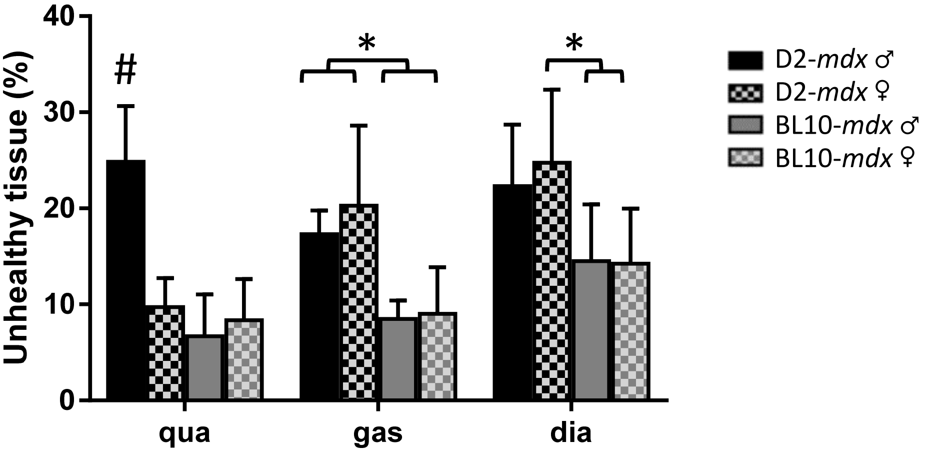

B

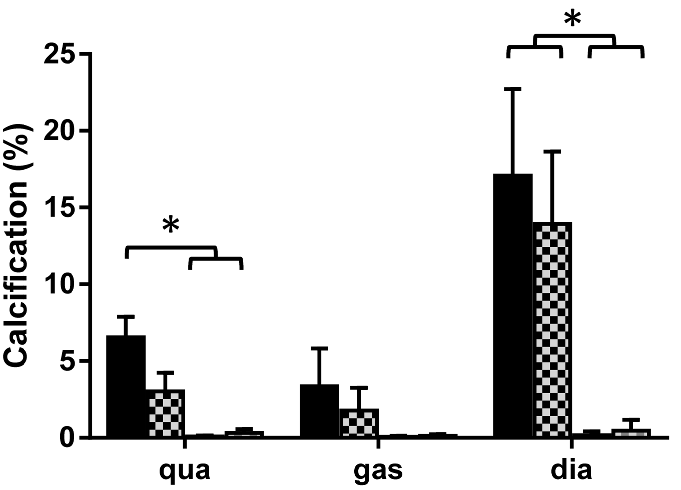

Supplementary figure 1. Histopathology differs between strains but not between genders.
A) Percentage unhealthy tissue assessed on H&E stained sections. Histopathology was more severe in the quadriceps of male D2-*mdx* mice compared to female counterparts. No gender differences were observed for the other muscles or the BL10-*mdx* strain. **B)** The percentage of calcification was pronounced in D2-*mdx* mice and almost absent in BL10-*mdx* mice, reaching significance in quadriceps and diaphragm. Although not significant, D2-*mdx* males had more calcification than females. *N*=6 mice per strain, per gender, # indicates a significant difference compared to all other groups. * indicates $P<0.05$. Values represent mean ± SD.
