# Supplementary material for: Natural disease history of the D2-mdx mouse model for Duchenne muscular dystrophy
Source: FASEB J. 2019 Apr 1;33(7):8110–24. doi: 10.1096/fj.201802488R (PMC6593893; doi:10.1096/fj.201802488R)
Supplement: Supplementary file 2 [file fj.201802488R.st1.docx]

**Table 1. Primer sequences used for qPCR analyses**

| **Gene** | **Function** | **Direction** | **3’-5’ sequence** |
| --- | --- | --- | --- |
| *Gapdh* | Housekeeping gene | Forward | TCCATGACAACTTTGGCATTG |
|  |  | Reverse | TCACGCCACAGCTTTCCA |
| *Col1a1* | Involved in fibrosis | Forward | ATGTTCAGCTTTGTGGACCT |
|  |  | Reverse | CAGCTGACTTCAGGGATGT |
| *Ctgf* | Involved in fibrosis, cell adhesion and proliferation. | Forward | AGCTGGGAGAACTGTGTACG |
|  |  | Reverse | GCCAAATGTGTCTTCCAGTC |
| *Lgals3* | Involved in inflammation, cell adhesion and growth and fibrosis | Forward | CAACCATCGGATGAAGAACC |
|  |  | Reverse | TTCCCACTCCTAAGGCACAC |
| *Cd68* | Involved in inflammation (macrophages) | Forward | CCAATTCAGGGTGGAAGAAA |
|  |  | Reverse | GAGAGAGACAGGTGGGGATG |
| *Ppar-γ* | Regulates fatty acid storage, glucose metabolism and adipogenesis | Forward | GACCAGGGAGTTCCTCAAAA |
|  |  | Reverse | CAGGTTGTCTTGGATGTCCTC |
| *Bmp2* | Involved in bone and cartilage development | Forward | GCAGCTTCCATCACGAAGA |
|  |  | Reverse | TCGTCACTGGGGACAGAACT |
| *Bmpr2* | Receptor for BMPs which are involved in osteogenesis | Forward | TAACTACCACCCCACCCTCA |
|  |  | Reverse | CAGAAACTGATGCCAAAGCA |
| *Ank* | Required for osteogenic differentiation of adult mesenchymal precursor cells | Forward | GGCATCACCATAGCCATC |
|  |  | Reverse | TCTACTGCATCCTCCTTGACTG |
| *Lrp5* | Key role in skeletal homeostasis | Forward | CCGAGGGAGCCTTTCTACTC |
|  |  | Reverse | CCCTGTCTTGCACGTCTTG |
| *Lrp6* | Co-receptor of Lrp5 | Forward | TGGCTCAAGTCGCTTCGTTAT |
|  |  | Reverse | TTCCGCTCCTGATAGTCCAGA |
| *Acvr1* | Involved in BMP pathway, responsible for development of the skeletal system | Forward | GTGGAAGATTACAAGCCACCA |
|  |  | Reverse | GGGTCTGAGAACCATCTGTTAGG |
| *Myod* | Involved in muscle differentiation | Forward | AGCACTACAGTGGCGACTCA |
|  |  | Reverse | GCTCCACTATGCTGGACAGG |
| *Myog* | Involved in muscle differentiation | Forward | CCCAACCCAGGAGATCATTT |
|  |  | Reverse | GTCTGGGAAGGCAACAGACA |
| *Mstn* | Inhibits myogenesis | Forward | CTCAGACCCGTCAAGACTCC |
|  |  | Reverse | CCTGGGCTCATGTCAAGTTT |
| *Stat3* | Involved in cell growth, | Forward | GCTGCTGCATCTTCTGTCTG |
|  |  | Reverse | TGAAGGTGGTGGAGAACCTC |
| *Serca2a* | Involved in calcium-transportation | Forward | ACCTGGAACAACCCGCAATAC |
|  |  | Reverse | CCCAACCTCAGTCATGCAGAG |
| *Vegf* | Involved in vasculogenesis and angiogenesis | Forward | CAGGCTGCTGTAACGATGAA |
|  |  | Reverse | GCATTCACATCTGCTGTGCT |
| *Nppa* | Controls extracellular fluid volumes and electrolyte homeostasis | Forward | CTGCAACAGCTTCCGGTACC |
|  |  | Reverse | GCTGCGTGACACACCACAAG |
